# Retraction: lncRNA FEZF1-AS1 Is Associated with Prognosis in Lung Adenocarcinoma and Promotes Cell Proliferation, Migration, and Invasion

**DOI:** 10.32604/or.2025.065346

**Published:** 2025-04-18

**Authors:** Oncology Research Editorial Office

The published article titled “lncRNA FEZF1-AS1 Is Associated with Prognosis in Lung Adenocarcinoma and Promotes Cell Proliferation, Migration, and Invasion” has been retracted from *Oncology Research*, Vol. 27, No. 1, 2019, pp. 39–45.

DOI: 10.3727/096504018X15199482824130

URL: https://www.techscience.com/or/v27n1/48658

The authors requested this retraction, informing the Editorial Office that some critical errors were presented in Fig. 2, Fig. 3 and Fig. 4, due to improper copying and reversal of the figure elements, duplication with figures published in other articles.

Following a review by the Editorial Board, the reasons provided for the retraction were found to be valid and detailed. All authors agreed to the retraction, and it was subsequently approved by the Editor-in-Chief.

As a responsible publisher, we hold the reliability and integrity of our published content in high regard. We deeply regret any inconvenience caused by this situation to our readers and all concerned parties.

